# Associations between recorded loneliness and adverse mental health outcomes among patients receiving mental healthcare in South London: a retrospective cohort study

**DOI:** 10.1007/s00127-024-02663-9

**Published:** 2024-04-15

**Authors:** Mayur Parmar, Ruimin Ma, Sumudu Attygalle, Maaheshi Deepika Herath, Christoph Mueller, Brendon Stubbs, Robert Stewart, Gayan Perera

**Affiliations:** 1 Department of Psychological Medicine, Institute of Psychiatry, Psychology and Neuroscience (King’s College London), De Crespigny Park, Box 92, London, SE5 8AF UK; 2 University of London, London, UK; 3 Ministry of Health Sri Lanka, Colombo, Sri Lanka; 4 South London and Maudsley NHS Foundation Trust, London, UK; 5 Faculty of Life and Health Sciences, School of Medicine, Ulster University, Belfast, Northern Ireland

**Keywords:** Loneliness, Mental disorders, Crisis episode, Mortality, Contacts with mental health services

## Abstract

**Purpose:**

Loneliness disproportionately affects people with mental disorders, but associations with mental health outcomes in groups affected remain less well understood.

**Method:**

A cohort of patients receiving mental healthcare on 30th June 2012 was assembled from a large mental health records database covering a south London catchment area. Recorded loneliness within the preceding 2 years was extracted using natural language processing and outcomes were measured between 30th June 2012 until 30th December 2019, except for survival which applied a censoring point of 6th December 2020 according to data available at the time of extraction. The following mental healthcare outcomes: (i) time to first crisis episode; (ii) time to first emergency presentation; (iii) all-cause mortality; (iv) days active to service per year; and (v) face-to-face contacts per year.

**Results:**

Loneliness was recorded in 4,483 (16.7%) patients in the study population and fully adjusted models showed associations with subsequent crisis episode (HR 1.17, 95% CI 1.07–1.29), emergency presentation (HR 1.30, 1.21–1.40), days active per year (IRR 1.04, 1.03–1.05), and face-to-face contacts per year (IRR 1.28, 1.27–1.30). Recorded loneliness in patients with substance misuse problems was particularly strongly associated with adverse outcomes, including risk of emergency presentation (HR 1.68, 1.29–2.18) and mortality (HR 1.29, 1.01–1.65).

**Conclusion:**

Patients receiving mental healthcare who are recorded as lonely have a higher risk of several adverse outcomes which may require a need for higher service input.

**Supplementary Information:**

The online version contains supplementary material available at 10.1007/s00127-024-02663-9.

## Background

Social relationships are known to have an effect on health, particularly when they are viewed to be positive [1]. Several publications have reported that there is a protective health effect of having a strong support network, but there is also a negative impact of their absence [2]. An absence of a support network can manifest in different forms, such as social isolation or loneliness. Social isolation occurs when an individual objectively has no, or very few satisfying relationships [3]. However, some individuals may find this circumstance to be adequate for their social needs. Loneliness, on the other hand, has been defined as distress arising from a mismatch in the quantity and/or quality of satisfying relationships compared to the desired level [4]. The prevalence of UK adults reporting feeling lonely often or always increased from 5% in 2018 [5] to 7% in 2021 [6]. Given research highlighting the deleterious effects of loneliness on physical and mental health, there is a need for it to be considered and addressed as a public health priority [7].

Disproportionately high prevalences of loneliness have been reported in patients with mental disorders [8]. Few studies have assembled samples comprised exclusively of people with mental disorders, but there is evidence suggesting that people who are lonely in these groups may have worse health outcomes. In a sample where 30.6% of participants were considered to have severe loneliness, Wang et al. (2020) found that a higher score of loneliness at baseline predicted poorer health-related quality of life four months later amongst a sample of people with mental disorders who received treatment from community crisis services [9]. Persistent loneliness was also associated with poor recovery 18 months later in patients with mental disorders [10]. In a sample of mental health service users in South London, Parmar et al. (2021) found that those who were recorded as being lonely were at a higher risk of acute general hospitalisation [11]. Recent studies found that loneliness was associated with increase in health service use including General Practitioner (GP) visits, Emergency Department (ED) visits and inpatient admissions but no significant association with attendance at ED or outpatient services, or for home visits among patients with psychosis [12]. Furthermore, certain diagnostic groups have been found to be associated with higher level of loneliness; for example, one study in London reported high level of loneliness among secondary mental health care users with common mental disorders and personality disorders [13].

Current evidence, while limited in quantity, is consistent in findings that mental health outcomes in samples of patients receiving mental healthcare are worse in patients self-identifying or described as lonely. Such outcomes include admission to psychiatric hospital in patients with severe mental illness [14, 15], and risk of non-accidental harm, depressed mood, psychotic symptoms, relationship problems and antidepressant use in older adults [16]. However, evidence in this field remains limited. Our study aimed to investigate recorded loneliness and its associations with mental health outcomes within patients receiving mental healthcare, drawing on data from a large service provider.

## Methods

### Study setting and data source

Data from the South London and Maudsley NHS Foundation Trust (SLaM) Biomedical Research Centre (BRC) Case Register were used to assemble this retrospective cohort study. SLaM is one of the largest mental healthcare providers in Europe, delivering services to a catchment area of over 1.3 million residents in the London boroughs of Lambeth, Lewisham, Croydon and Southwark [17]. SLaM’s Clinical Record Interactive Search (CRIS) data resource was developed with National Institute for Health Research funding in 2007-8. CRIS provides researcher access to deidentified electronic health records data on over 500,000 cases within a robust government framework [18] and has supported extensive research output [19, 20]. The electronic health records contain structured and unstructured (free) text fields and CRIS data have been extensively supplemented by the use of natural language processing (NLP) algorithms to ascertain constructs within the latter [17]. Data enhancements have also been achieved through linkages to other databases, including Hospital Episode Statistics (HES) which contain statistical abstracts of all inpatient, outpatient and emergency care records from all healthcare providers in England [21]. CRIS and its linkages, including HES, have full approval for secondary analysis (Oxford Research Ethics Committee C, reference 18/SC/0372).

### Sample

This study assembled a cohort from the SLaM BRC Case Register using CRIS to capture changes over time. The studied population comprised patients who had an ‘active’ SLaM record on the index date of 30th June 2012 (i.e., had a referral that had been accepted and were not discharged from services on that dated). This sample was assembled to describe the prevalence of recorded loneliness and ascertain outcome associations.

### Exposure

The primary exposure of interest was loneliness described as experienced by the patient in question in the electronic health record. Importantly, there is no routinely completed structured field in the health record for this construct. Therefore, we searched for relevant words in text fields and applied an NLP algorithm, developed using Generalised Architecture for Text Engineering (GATE) and TextHunter software [22]. In preparation for this project, initial keyword searches had been carried out in CRIS to explore terminology used by clinical staff to describe loneliness in the source records and the terms “lonely” and “loneliness” were found to give the most common description of the experience we aimed to measure. A decision was made to focus on ascertaining this construct initially, although the intention is ultimately to broaden this to ascertaining recorded levels of social support – an overlapping but not identical entity. The accuracy of the algorithm for identifying recorded loneliness in records (i.e., loneliness recorded as present rather than absent or hypothetical, and applying to the patient rather than anyone else) was found to be high (precision = 87%, recall = 100%) on independent checks of 100 records by two annotators (Cohen’s kappa = 81%). A comprehensive summary of algorithms in current use can be found in the SLaM NLP Applications Library [23], and the development of the NLP algorithm for loneliness has been described in detail in Parmar et al., (2021) [11]. Recorded loneliness identified by the NLP algorithm at any point on or within two years prior to the index date was defined as the exposure.

### Covariates

Covariates obtained at the index date were age, gender, ethnicity (White, Asian, Black, Mixed, or Other). Other covariates were measured between one year before and three months after the index date and, if multiple values had been recorded, the value closest in time to the index date was used. These included binary variables on cohabiting status (cohabiting – married, cohabiting; not cohabiting – single, divorced, widowed), and a mean score on the Index of Multiple Deprivation (IMD; 2015), a measure of area-level socioeconomic status containing information from seven domains (income; employment; health and disability; education, skills and training; barriers to housing and services; crime; and living environment) derived from national Census data [24]. The measure is applied to Lower Super Output Areas (LSOAs) within the SLaM catchment, administrative small areas of 1500–2000 residents. A higher mean score on IMD indicates greater level of deprivation. Health of the Nation Outcome Scales (HoNOS) are 4-point measures of health and social functioning that are routinely administered in mental health services in the UK. HoNOS items include agitation, self-injury, substance use problems, cognitive problems, physical health problems, hallucinations/delusions, depression, relationship problems, daily living problems, living conditions problems and occupational problems [25]. These subscale scores were extracted as structured data directly from the electronic health record. Primary psychiatric diagnosis closest to index date was assigned from structured ICD-10 codes, and the following specific groups were ascertained: severe mental illness (SMI; F2*, F31*) mental and behavioural disorders due to psychoactive substance use (F10*-F19*), dementia (F0*-F03*), anxiety (F40*-F42*), personality disorders (F60*-F61*) eating disorders (F50*), and depression (F32*-F33*). Medication use was extracted from an NLP algorithm applied to text fields to generate the following binary covariates: anticoagulants, antihypertensives, diabetic medication, beta-blockers, analgesics, antidepressants, antihypertensives, anxiolytics and hypnotics. Data were obtained from the linked HES tables on admissions (emergency or elective) to general hospitals. Emergency admissions are defined under codes 21–24 and 28, and elective admissions under codes 11–13 within ‘admission methods’ within the NHS data dictionary [26]. The number of emergency and elective admission to general hospital were included as separate covariates, and binary variables were generated for the occurrence of admissions with a primary discharge diagnosis under the following ICD-10 chapters: genitourinary (chapter N), musculoskeletal (M), digestive (K), respiratory (J) and circulatory (I). The total number of documents available for NLP as a proxy for frequency and level of contact until index date was measured.

### Outcomes

Outcomes were measured between 30th June 2012 until 30th December 2019, except for survival which applied a censoring point of 6th December 2020 according to data available at the time of extraction. The following outcomes were derived: **(i)** time to first crisis episode, defined as time to first inpatient or home treatment team (HTT) episodes after the index date; **(ii)** time to first emergency care presentation, defined as first contact with a catchment psychiatric liaison service contact in an emergency room setting after index date; **(iii)** time to date of death (i.e., all-cause mortality); **(iv)** mean number of days active in SLaM per year, defined as the total number of days within outpatient and/or inpatient spells of care during the follow-up period divided by the number of years of follow-up from the index date to 31st December 2019 or date of death, whichever was earliest; **(v)** number of face-to-face contacts per year, measured as outpatient and community contacts only, as recorded in structured fields for all entered encounters (and excluding inpatient, telephone or other virtual contacts), with number of years follow-up also calculated as a denominator from the index date to 31st December 2019 or the date of death.

### Statistical analysis

Initially, descriptive characteristics of patients recorded as lonely were compared with the remainder of patients. Cox proportional hazard models (generating hazard ratios; HR) were carried out to investigate associations of loneliness with time to crisis episode, emergency presentation and survival. Poisson regression analyses (generating incidence rate ratios; IRR) were conducted to investigate the associations between recorded loneliness and number of days active per year, and number of face-to-face contacts per year. Models were adjusted for number of documents available to review, sociodemographic variables, psychotropic medications and psychiatric diagnoses, physical health conditions and medications for physical health. Analyses were also stratified by primary psychiatric diagnosis at the time of index date to visualise the extent of homogeneity across diagnoses. Supplementary analyses were carried out to investigate association between loneliness and mental health outcomes adjusted for each HoNOS symptoms only for patients with HoNOS data and exploratory stratification by age and gender with likelihood ratio tests of respective interaction terms.

## Results

Table 1 displays the characteristics of the study cohort. On the index date of 30th June 2012, 4,483 (16.7%) patients out of a total of 26,737 had been recorded as lonely. Statistical significance tests were not carried out, but it should be noted that patients with recorded loneliness were older and were living in higher-deprivation neighbourhoods and recorded loneliness was over-represented in women, most minority ethnic groups, and in those living alone or non-cohabiting. HoNOS items, where present, showing higher problems in those with recorded loneliness included self-injury, substance use, hallucinations, depressed mood, relationships, and living conditions. The group with recorded loneliness were also more likely to have SMI, depression, anxiety, eating disorder and personality disorder diagnoses, and all medication groups analysed were higher in this group, as were general hospital admissions; higher numbers of documents within the record were also observed. The mean (SD) duration of loneliness from the recorded loneliness date to either date of death or 31st March 2019 (whichever was earliest) was 0.67 (2.10) years for cohabiting patients and 1.34 (2.83) years for non-cohabiting patients.

**Table 1:** Characteristics of the study cohort by baseline recorded loneliness

	Number (%) or mean (SD)	
	Recorded as lonely (*n* = 26,737)	
Characteristics	No (*n* = 22,254)	Yes (*n* = 4,483)
Mean (SD) age (SD)	46.4 (18.4)	47.9 (18.7)
*Gender*		
Female	10,497 (47.2)	2,501 (55.8)
Male	11,757 (52.8)	1,981 (44.2)
*Ethnicity*		
White	14,222 (63.9)	2,631 (58.7)
Asian	1,275 (5.7)	292 (6.5)
Black	4,688 (21.1)	1231 (27.5)
Mixed	541 (2.4)	116 (2.6)
Other	1399 (6.3)	210 (4.7)
Living alone	4304 (19.3)	1429 (31.9)
*Cohabiting status*		
Cohabiting	5556 (25.0)	584 (13.0)
Not cohabiting	16,698 (75.0)	3899 (87.0)
Mean (SD) area-level deprivation (IMD 2015)	28.6 (11.2)	30.4 (10.1)
Number of patients with HoNOS data	10,294 (46.3)	3,261 (72.7)
*HoNOS problems closest to index date* ^$^		
Agitated Behaviour	1,707 (16.6)	501 (15.4)
Self-Injury	551 (5.4)	262 (8.0)
Drinking Drugs	1,152 (11.2)	443 (13.6)
Cognitive Problems	2,767 (26.9)	711 (21.8)
Physical Illness	3,267 (31.7)	1,057 (32.4)
Hallucinations	2,314 (22.5)	792 (24.3)
Depressed Mood	3,484 (33.8)	1,232 (37.8)
Relationship Problems	3,225 (31.3)	1,278 (39.2)
Daily Living Problems	3,507 (34.1)	1,110 (34.0)
Living Conditions Problems	1,385 (13.5)	524 (16.1)
Occupational Problems	2,888 (28.1)	965 (29.6)
*Psychiatric diagnosis closest to index date*		
Dementia (F0- F03)	2133 (9.6)	430 (9.6)
Psychoactive substance misuse (F10- F19)	2384 (10.7)	486 (10.8)
SMI (F20- F29 and F31)	5366 (24.1)	1840 (41.0)
Depression (F32-F33)	3021 (13.6)	1035 (23.1)
Anxiety (F40- F42)	1154 (5.2)	284 (6.3)
Eating disorders (F50)	299 (1.3)	120 (2.7)
Personality disorders (F60- F61)	663 (3.0)	384 (8.6)
*Medication closest to index date*		
Anticoagulants	436 (2.0)	171 (3.8)
Diabetic medication	1014 (4.6)	351 (7.8)
Beta blockers	704 (3.2)	257 (5.7)
Analgesics	3,847 (17.3)	1046 (23.3)
Antidepressants	8,402 (37.8)	2,547 (56.8)
Antihypertensives	2,341 (10.5)	704 (15.7)
Anxiolytics and hypnotics	5,876 (26.4)	2,066 (46.1)
*Hospital admissions closest to index date*		
Mean number of elective admissions (SD)	0.4 (3.9)	0.5 (4.6)
Mean number of emergency admissions (SD)	0.3 (1.1)	0.7 (1.7)
Genitourinary	1107 (5.0)	305 (6.8)
Musculoskeletal	1147 (5.2)	288 (6.4)
Digestive	1687 (7.6)	417 (9.3)
Respiratory	1267 (5.7)	365 (8.1)
Circulatory	1902 (8.5)	522 (11.6)
Mean number of documents (SD)	85.1 (221.0)	279.6 (441.0)

Table 2 summarises the distribution of outcomes of the study and the multivariable models for each of the associations are displayed in Table 3. Fully adjusted models showed associations of recorded loneliness with increased likelihood of a crisis episode (HR 1.17, 95% CI 1.07–1.29, *p* < 0.001), an emergency presentation (HR 1.30, 95% CI 1.21–1.40, *p* < 0.001), more days active in SLaM per year (IRR 1.04, 95% CI 1.03–1.05, *p* < 0.001), and more face-to-face contacts per year (IRR 1.28, 95% CI 1.27–1.30, *p* < 0.001). An association with higher mortality was present in Model 1 but this was not apparent in any adjusted model.

**Table 2:** Outcomes in the study cohort by baseline recorded loneliness

	Number (%) or mean (SD)	
	Recorded as lonely (*n* = 26,737)	
Outcome	No (*n* = 22,254)	Yes (*n* = 4,483)
Any crisis episode	4,272 (19.2)	1,687 (37.7)
Any emergency presentation	6,834 (30.7)	2,065 (46.1)
Mean number of attended face to face contacts per year	5.7 (9.6)	11.1 (13.8)
Mean number of days active in SLaM per year	151.2 (206.6)	185.6 (129.3)
Number deceased	4,201 (18.9)	968 (21.6)
Mean time to first crisis episode	1.1 (0.9)	0.7 (0.5)
Mean time to first emergency presentation	0.9 (0.6)	0.6 (0.5)
Mean time to death	3.9 (2.4)	4.1 (2.4)

**Table 3:** Regression analyses of associations of recorded loneliness with mental health outcomes. Regression outputs are displayed with 95% confidence intervals and p-values

	Crisis episode (HR)	Emergency presentation (HR)	Mortality (HR)	Number of days active in SLaM per year (IRR)	Number of face-to-face contacts per year (IRR)
Regression analysis	Cox proportional hazards	Cox proportional hazards	Cox proportional hazards	Poisson	Poisson
Model 1: (*n* = 26,737)	1.41 (1.30, 1.53), < 0.001	1.77 (1.65, 1.89), < 0.001	1.14 (1.06, 1.22), < 0.001	1.11 (1.11, 1.12), < 0.001	1.63 (1.61, 1.65), < 0.001
Model 2: (*n* = 26,605)	1.39 (1.28, 1.51), < 0.001	1.69 (1.57, 1.81), < 0.001	0.98 (0.90, 1.04), 0.31	1.09 (1.09, 1.10), < 0.001	1.54 (1.53, 1.56), < 0.001
Model 3: (*n* = 26,605)	1.17 (1.07, 1.28), < 0.001	1.32 (1.22, 1.42), < 0.001	0.96 (0.90, 1.04), 0.31	1.04 (1.04, 1.05), < 0.001	1.29 (1.27, 1.30), < 0.001
Model 4: (*n* = 26,605)	1.17 (1.07, 1.29), < 0.001	1.30 (1.21, 1.40), < 0.001	0.93 (0.86, 1.01), 0.07	1.04 (1.03, 1.05), < 0.001	1.28 (1.27, 1.30), < 0.001

^Model 1 adjusted for number of documents available to review; model 2: model 1 + sociodemographic; model 3: model 2 + psychiatric medication and conditions; model 4: model 3 + physical health medication and conditions^

Table 4 summarises study outcomes according to the primary psychiatric diagnoses evaluated. Particularly, patients who were recorded lonely were more like to have worse mental health outcomes among patients whose primary diagnosis was psychoactive substance misuse. Recorded loneliness in patients with SMI was associated with significantly worse outcomes for crisis episodes, emergency presentations, and more face-to-face contacts per year. Recorded loneliness in patients with eating disorders was associated with increased risk of crisis episodes, more days active per year and more face-to-face contacts per year.

**Table 4:** Adjusted* associations of recorded loneliness with mental health outcomes within diagnostic groups. Regression outputs are displayed with 95% confidence intervals and p-values

Psychiatric diagnosis	Crisis episode (HR)	Emergency presentation (HR)	Mortality (HR)	Number of days active in SLaM per year (IRR)	Number of face-to-face contacts per year (IRR)
Dementia (F00x-03x)	1.00 (0.59, 1.70), 0.99	1.50 (1.09, 2.07), 0.01	0.84 (0.74, 1.03), 0.08	0.98 (0.92, 1.03), 0.22	1.27 (1.20, 1.35), < 0.001
Substance use disorders (F1x)	1.23 (1.02, 1.55), 0.04	1.68 (1.29, 2.18), < 0.001	1.29 (1.01, 1.65), 0.04	1.06 (1.04, 1.08), < 0.001	1.23 (1.18, 1.27), < 0.001
SMI (F2x, F31x)	1.27 (1.11, 1.45), < 0.001	1.26 (1.12, 1.42), < 0.001	0.86 (0.74, 1.02), 0.06	1.01 (0.99, 1.02), 0.43	1.17 (1.16, 1.19), < 0.001
Depression (F32x-33x)	0.95 (0.74, 1.24), 0.72	1.32 (1.08, 1.62), 0.01	1.00 (0.79, 1.25), 0.97	1.12 (1.11, 1.13), < 0.001	1.41 (1.36, 1.47), < 0.001
Anxiety (F40x-42x)	1.11 (0.65, 1.90), 0.70	0.92 (0.62, 1.37), 0.68	0.91 (0.54, 1.54), 0.73	0.95 (0.88, 1.02), 0.18	1.24 (1.13, 1.37), < 0.001
Eating disorders (F50x)	1.14 (1.04, 1.87), 0.04	1.23 (0.67, 2.26), 0.50	2.00 (0.35, 11.56), 0.44	1.12 (1.09, 1.15), < 0.001	1.62 (1.50, 1.76), < 0.001
Personality disorders (F60x-F61x)	0.83 (0.56, 1.22), 0.34	1.22 (0.87, 1.71), 0.26	0.68 (0.42, 1.11), 0.12	1.01 (0.94, 1.04), 0.54	1.29 (1.23, 1.35), < 0.001

^*Adjusted for number of documents available to review, sociodemographic factors, psychiatric medication and conditions, physical health medication and conditions^

When patients with available HoNOS data were analysed, adjusting for each HoNOS component (**Online Resource 1**) outcome associations did not change substantially following adjustment. When stratified by age and gender (**Online Resource 2**), most associations showed overlapping confidence intervals apart from associations with increased number of days active per year, which were stronger in older and female patients, and associations with increased face-to-face contacts per year, which were stronger in younger and female patients. Within older age groups, loneliness prevalence was slightly higher among 80 + year-olds (20.4%) compared with 65-79-year-olds (18.2%). Applying likelihood ratio tests for crisis episode as an outcome, no interactions were found with gender (chi-squared (df), p-value: 0.05 (1), 0.83) or age group (3.57 (4), 0.47). Applying likelihood ratio tests for mortality as an outcome, no interaction was found with age group (5.18 (4), 0.27) but an interaction was close to significance for gender (3.55 (1), 0.060).

## Discussion

Our study investigated the recording of loneliness within a large cohort of patients receiving mental healthcare from a south London catchment, evaluating this construct and its associated outcomes based on its presence within 2 years up to a specific census date. Our study estimated a point prevalence of 16.7% for recorded loneliness, and this definition of recorded loneliness was associated prospectively with increased risk of crisis episode and emergency presentation, and with higher proportions of time in receipt of mental healthcare and contacts with mental healthcare staff. Loneliness was associated with higher mortality in unadjusted analyses but not in adjusted models.

We found patients who were recorded as lonely were more likely to have subsequent crisis episodes than the remainder of the sample. In the general population, loneliness is associated with more visits to a physician as well as higher frequency of inpatient treatments [27]. We were able to generalise this finding to mental health service provision as well as to cohorts exclusively made up of people receiving mental healthcare, thus increasing its potential relevance for mental health services. When considering the few previous studies that have looked at psychiatric hospitalisation within a sample of people with mental disorders, they typically found a positive association with loneliness [14, 15, 28]. Our study added to this finding more broadly, with data on inpatient and outpatient crisis team use. Many studies do not consider the length of time patients have a mental health diagnosis when comparing outcomes of loneliness.

We also measured emergency care presentations resulting in review by local psychiatric liaison services, and out study showed increased risk of this outcome associated with recorded loneliness. A recent study found that a higher number of psychiatric hospitalisations including emergency presentations in the past 6 months were associated with greater loneliness [29], consistent with our finding. A qualitative study from Sweden also investigated reasons for frequent use of psychiatric emergency services, and cited loneliness as one of the key problem areas [30]. The professionals who were interviewed in this study reported that utilisation of psychiatric emergency services was frequent because it helped to relieve feelings of loneliness, staff in these services were familiar, and it gave patients an opportunity to have a personal conversation with someone who recognised their likes and dislikes. Another study suggested a sequence in people with long-term mental disorders of difficulty in establishing satisfying relationships, which increases loneliness, resulting in worse symptoms [31]. Evaluation of severity of symptoms could therefore be a fruitful line of future research in understanding the relationship with adverse outcomes in more detail.

The evidence base is currently mixed on the relationship of loneliness with mortality in the general population. In line with our own results, Steptoe et al., (2013) found that after adjusting for sociodemographic variables, loneliness no longer predicted mortality over 7 years of follow-up [32]. In contrast, other studies have reported an association with early mortality in a sample of people with SMI [33]. Poor health behaviours and low self-efficacy are commonly cited as contributing to early mortality experienced by people who are lonely [34]. There is a possibility that our follow-up was of insufficient duration, underlying the non-significant association. Furthermore, in people with mental health problems, their mortality may already have been severely impacted by their mental health conditions and associated with poor health behaviours and low self-efficacy; therefore, any effect of loneliness might be obscured by competing risks. Further studies may need to investigate loneliness in people receiving mental healthcare longitudinally over a longer period of time, as patterns of mortality can take up to several decades to observe.

Our study found that loneliness was associated with a higher proportion of time spent in receipt of mental health services. Studies that have investigated time spent in mental health services have rarely considered the impact of loneliness as a contributor, despite mounting evidence of associations with severity of mental and physical health [35]. The number of face-to-face contacts per year was significantly higher for patients who were recorded as lonely. This could be attributed to engagement with clinicians and therapists while in services. Cacioppo et al. (2006) proposed that loneliness stimulates social-seeking behaviours [36]. For people with mental disorders who already have a small social network, healthcare professionals are a viable next option, and could explain why face-to-face contacts are higher in our cohort.

Furthermore, our study showed potential variation in associations with mental health outcomes by primary diagnosis, although confidence intervals overlapped. In general, strongest associations with worse mental health outcomes tended to be observed among patients with primary substance use disorder diagnoses, those with SMI, and those with eating disorders. People with substance use problems have been found to report more loneliness than the general population [37] and previous studies have found that people who have substance misuse problems and who are lonely are at higher risk of low self-esteem and suicidality [38]. However, available interventions to reduce loneliness are limited in this group [38]. Loneliness has previously been reported as associated with worse outcomes for people with dementia [39], depression [40], anxiety [27] and psychosis [41]. US evidence indicates that individuals with substance use disorders often access the health care system for reasons other than their substance use disorder, with many not seeking specialty treatment but over-represented in general health care settings [42]. Mental health comorbidity is also recognised to be high in drug (70%) and alcohol (86%) users in community substance misuse treatment [43]. The National Confidential Inquiry into Suicide and Homicide by People with Mental Illness found that suicide-related deaths among people with a history of alcohol or drug use (or both) accounted for 54% of all such mortality [44]; however, only 11% of these people were in touch with alcohol or drug services at the time of death. Both alcohol and drug use and mental health problems are associated with considerable physical morbidity and premature mortality (15–20 years in people with mental health problems and 9–17 years in those with alcohol and drug use disorders) compared to national norms [45]. People with mental health problems are more likely to smoke and smoking is the single largest contributor to their 10-20-year reduced life expectancy.

Our study also found that most associations had overlapping confidence intervals when stratified by age group and gender, and most interaction tests did not approach statistical significance apart from that between loneliness and gender for mortality as an outcome. Mean ages were similar in our sample between those with/without recorded loneliness. One recent study reported that levels of loneliness were highest in 18-23-year-olds, followed by 24-39-year-olds, and lowest in 56-74-year-olds [46]; however, this used a screening instrument administered in an online survey during the initial period of the COVID-19 pandemic and comparability may therefore be limited. More generally, people receiving mental healthcare may well have different patterns and correlates of loneliness compared to community samples because of the profound impact a mental health condition may have on the social environment, as well as the cumulative influence of the social environment on risk of mental disorder.

Our findings are consistent with those from a survey carried out by the Mental Health Foundation where one in three Black people reported experiencing feelings of loneliness [47], compared to one in four of the general population reporting feeling lonely some or all of the time. That report highlighted racism and financial inequality as contributory factors. Furthermore, another recent study found that black and minority ethnic communities are disproportionately impacted by social challenges associated with mental ill health [48].

10% of our study cohort comprised patients with dementia. Previous studies have found that poor social engagement is associated with increased dementia risk, although loneliness specifically was not significantly associated [49]. On the other hand, loneliness has been reported to have negative impacts on cognitive decline [50] and dementia progression [51], although we did not seek to investigate this. At older ages, loneliness is also a major risk factor for broader morbidity outcomes both psychological and physical, including depressive symptoms, worse physical health, and functional limitations [52] as well as with diminished or poorer-quality sleep [53].

This study is among the first to utilise natural language processing to ascertain recorded loneliness at scale in routine healthcare records and we were thus able to evaluate its impact in a large, naturalistic sample of people attending mental health services. However, our findings should also be reviewed in light of some limitations. First, while NLP opens novel opportunities, it relies heavily on the quality of clinical notes within electronic health records. There is no current obligation for loneliness to be inquired about in routine clinical practice; therefore, it is likely that many patients who subjectively felt lonely did not bring it up with a clinician, or the clinician did not record it, resulting in an underestimated prevalence. The lack of guidelines surrounding loneliness also contribute to this limitation; without specific terminology being used to enter data to electronic patient records, we may have made further omissions to patients who felt lonely but did not use commonly used phrasing to describe their experience. For example, isolation and loneliness tend to be used interchangeably by clinicians despite them not capturing the same experience. Second, CRIS only contains patients from a single service provider and wider generalisability needs evaluation. The data on follow-ups may also miss people who out-migrate from the catchment. Third, as with many studies that investigate loneliness and health, causal relationships cannot be concluded with certainty from observational data. Loneliness may have resulted in the increased service use identified in this study, or possibly patterns of service use might influence loneliness. The direction of the effects found in this study should be established longitudinally.

Our findings suggest that people who are lonely have a higher risk of a range of adverse outcomes in mental healthcare, presenting potential implications for demand on service input and public health. Inquiring about loneliness is not routinely expected in clinical assessments, even in mental health services where it is a particularly pertinent factor. High prevalence of loneliness among service users is not only an individual but also a community and societal level problem, and macrosocial factors have been proposed as significant determinants of levels and content of social relationships [54]. Therefore, it is imperative to advance public awareness of the importance of reducing loneliness, and ultimately to urge policy level changes to support disadvantaged populations. While the topic can arise sporadically when a patient feels able to report it, it may be useful for the status of social circumstances to be a routine component of the clinical assessment. A validated short screening tool might potentially provide practitioners with a time- and cost-effective approach to identify people in need, for example, the De Jong Gierveld 6-Item Loneliness Scale [55] or the UCLA 3-Item Loneliness Scale [56]. Furthermore, improved clinical awareness of loneliness could increase the efficiency of signposting to services that specialise in alleviating loneliness within the voluntary sector or by social prescribing, although further research is needed to clarify the effectiveness of these interventions. Allowing loneliness to continue can contribute to healthcare use through increased and prolonged presentations, as well as costly crisis episodes. We present findings supporting this, and future studies should aim to further investigate the relevance of duration of loneliness, as well as social resources that might be drawn on to alleviate it and the role of health services in facilitating improvement.

## Electronic supplementary material

Below is the link to the electronic supplementary material.

Supplementary Material 1

## Data Availability

No additional data are available.

## Code availability

Codes not used.
