# Supplementary material for: Associations between recorded loneliness and adverse mental health outcomes among patients receiving mental healthcare in South London: a retrospective cohort study
Source: Soc Psychiatry Psychiatr Epidemiol. 2024 Apr 15;59(12):2155–64. doi: 10.1007/s00127-024-02663-9 (PMC11522161; doi:10.1007/s00127-024-02663-9)
Supplement: Supplementary file 1 — Supplementary Material 1 [file 127_2024_2663_MOESM1_ESM.docx]

**Online Resource 1: Associations of recorded loneliness with mental health outcomes further adjusted for individual HoNOS symptoms.**

| **Adjustment** | **Crisis episode (OR)** | **Emergency presentation (OR)** | **Mortality (HR)** | **Number of days active in SLAM per year (IRR)** | **Number of face-to-face contacts per year (IRR)** |
| --- | --- | --- | --- | --- | --- |
| Model 4 (Table 3) (n= 26,605) all patients | 1.17 (1.07, 1.29), <0.001 | 1.30 (1.21, 1.40), <0.001 | 0.93 (0.86, 1.01), 0.07 | 1.04 (1.03, 1.05), <0.001 | 1.28 (1.27, 1.30), <0.001 |
| Model 4 (Table 3) (n= 13,538) restricted to those with HoNOS data | 1.07 (0.96, 1.19), 0.21 | 1.19 (1.09, 1.30), <0.001 | 0.92 (0.85, 100), 0.06 | 1.03 (1.02, 1.04), <0.001 | 1.23 (1.21, 1.25), <0.001 |
| *Additional separate adjustments for HoNOS subscales* | |  |  |  |  |
| Agitated behaviour (n=13,518) | 1.07 (0.97, 1.19), 0.18 | 1.19 (1.09, 1.30), <0.001 | 0.93 (0.86, 1.02), 0.11 | 1.03 (1.02, 1.04), <0.001 | 1.23 (1.22, 1.25), <0.001 |
| Self-injury (n=13,518) | 1.07 (0.96, 1.19), 0.22 | 1.18 (1.08, 1.29), <0.001 | 0.92 (0.85, 1.00), 0.06 | 1.03 (1.02, 1.04), <0.001 | 1.23 (1.22, 1.25), <0.001 |
| Substance use (n=13,518) | 1.07 (0.96, 1.19), 0.22 | 1.19 (1.09, 1.30), <0.001 | 0.91 (0.84, 1.00), 0.06 | 1.03 (1.02, 1.04), <0.001 | 1.24 (1.22, 1.25), <0.001 |
| Cognitive problems (n=13,518) | 1.06 (0.96, 1.18), 0.24 | 1.18 (1.08, 1.29), <0.001 | 0.94 (0.87, 1.02), 0.16 | 1.03 (1.02, 1.04), <0.001 | 1.23 (1.22, 1.25), <0.001 |
| Physical illness (n=13,518) | 1.07 (0.96, 1.19), 0.23 | 1.19 (1.09, 1.30), <0.001 | 0.93 (0.85, 1.01), 0.07 | 1.03 (1.02, 1.04), <0.001 | 1.23 (1.22, 1.25), <0.001 |
| Hallucinations (n=13,518) | 1.08 (0.97, 1.19), 0.17 | 1.18 (1.08, 1.29), <0.001 | 0.93 (0.86, 1.01), 0.10 | 1.03 (1.02, 1.04), <0.001 | 1.24 (1.22, 1.26), <0.001 |
| Depressed mood (n=13,518) | 1.08 (0.97, 1.19), 0.17 | 1.19 (1.09, 1.30), <0.001 | 0.92 (0.85, 1.01), 0.07 | 1.03 (1.02, 1.04), <0.001 | 1.24 (1.22, 1.25), <0.001 |
| Relationship problems (n=13,518) | 1.07 (0.97, 1.19), 0.20 | 1.19 (1.09, 1.30), <0.001 | 0.92 (0.85, 1.00), 0.06 | 1.03 (1.02, 1.04), <0.001 | 1.23 (1.22, 1.25), <0.001 |
| Daily Living problems (n=13,518) | 1.07 (0.96, 1.19), 0.21 | 1.19 (1.09, 1.30), <0.001 | 0.95 (0.88, 1.04), 0.27 | 1.03 (1.02, 1.04), <0.001 | 1.24 (1.22, 1.25), <0.001 |
| Living Conditions problems (n=13,518) | 1.07 (0.97, 1.19), 0.19 | 1.19 (1.09, 1.30), <0.001 | 0.92 (0.85, 1.00), 0.06 | 1.03 (1.02, 1.04), <0.001 | 1.24 (1.22, 1.25), <0.001 |
| Occupational problems (n=13,518) | 1.07 (0.97, 1.19), 0.19 | 1.19 (1.09, 1.30), <0.001 | 0.92 (0.85, 1.00), 0.06 | 1.03 (1.02, 1.04), <0.001 | 1.23 (1.22, 1.25), <0.001 |

**Online Resource 2: Associations of recorded loneliness with mental health outcomes stratified by age and gender.**

| **Stratification** | **Crisis episode (OR)** | **Emergency presentation (OR)** | **Mortality (HR)** | **Number of days active in SLAM per year (IRR)** | **Number of face-to-face contacts per year (IRR)** |
| --- | --- | --- | --- | --- | --- |
| Model 4 (Table 3) (n= 26,605) | 1.17 (1.07, 1.29), <0.001 | 1.30 (1.21, 1.40), <0.001 | 0.93 (0.86, 1.01), 0.07 | 1.04 (1.03, 1.05), <0.001 | 1.28 (1.27, 1.30), <0.001 |
| **By age** |  |  |  |  |  |
| Over 60 (n= 5,885) | 1.24 (1.01, 1.53), 0.04 | 1.38 (1.18, 1.62), <0.001 | 0.92 (0.82, 1.02), 0.07 | 1.06 (1.05, 1.06), <0.001 | 1.17 (1.14, 1.21), <0.001 |
| Under 60 (n= 20,720) | 1.15 (1.04, 1.28), 0.01 | 1.29 (1.19, 1.40), <0.001 | 0.99 (0.87, 1.14), 0.93 | 1.01 (1.01, 1.01), <0.001 | 1.31 (1.29, 1.33), <0.001 |
| **By gender** |  |  |  |  |  |
| Male (n= 13,685) | 1.13 (0.99, 1.29), 0.07 | 1.37 (1.22, 1.52), <0.001 | 0.83 (0.70, 1.01), 0.06 | 1.03 (1.03, 1.04), <0.001 | 1.21 (1.19, 1.23), <0.001 |
| Female (n= 12,919) | 1.19 (1.05, 1.35), 0.01 | 1.25 (1.13, 1.38), <0.001 | 1.00 (0.91, 1.10), 0.98 | 1.05 (1.05, 1.06), <0.001 | 1.34 (1.32, 1.36), <0.001 |
